# Geographic variation in the clinical features of Mohave rattlesnake (*Crotalus scutulatus*) envenomations reported to the North American Snakebite Registry

**DOI:** 10.1016/j.toxcx.2023.100171

**Published:** 2023-11-10

**Authors:** Spencer Greene, Matthew Gilbert, Brian Wolk, Sharan Campleman, Anne-Michelle Ruha, on behalf of the ToxIC Snakebite Study Group

**Affiliations:** a University of Houston/HCA Kingwood Emergency Medicine Residency Program, Kingwood, TX, USA; b Department of Emergency Medicine, Loma Linda University School of Medicine, Loma Linda, CA, USA; c American College of Medical Toxicology, Phoenix, AZ, USA; d Department of Medical Toxicology, Banner University Medical Center – Phoenix, Phoenix, AZ, USA

**Keywords:** Antivenom, Myotoxin, Neurotoxicity, Phospholipases, Pitviper, Rattlesnake

## Abstract

The geographic variation of Mohave rattlesnake (*Crotalus scutulatus*) venom is well established. We reviewed all the Mohave rattlesnake bites reported to the Toxicology Investigators Consortium's North American Snakebite Registry between January 1, 2015 and 12/31/2021. Data reviewed for this study included details regarding the snake encounter, patient demographics, signs and symptoms, treatment, and outcomes. Our objective was to describe the epidemiology, clinical manifestations, and management of Mohave rattlesnake envenomations using prospective data from two geographically distinct sites. There were 20 subjects, including eight nonpregnant females. Ages ranged from seven to 75 years, median age 48. Nine of the bites were managed in Arizona and 11 in California. In Arizona, all envenomated patients had local swelling. None had neurological toxicity. In California, swelling was present in nine patients. Neurological effects were observed in five subjects. Four Arizona patients and one California patient had hypotension requiring treatment. Each site had one patient with thrombocytopenia. An Arizona patient who sustained a bite to the face was intubated. Rhabdomyolysis occurred in two California patients. All envenomated patients received antivenom. Mohave rattlesnakes have the potential to cause significant local and/or systemic toxicity. Neurotoxicity was not observed in envenomations from Mohave rattlesnakes that presumably lack Mohave toxin, but hypotension and gastrointestinal signs were more common than in bites from snakes believed to possess Mohave toxin. Neurological toxicity was limited to paresthesias and fasciculations. Significant skeletal or respiratory muscle weakness was not observed in our study population.

## Highlights

• Venom components of Mohave rattlesnake vary geographically.
• Mohave rattlesnakes possessing Mohave toxin are found in California, Nevada, New Mexico, Utah, and southeastern Arizona.
• Mohave toxin inconsistently causes neurological toxicity.
• Mohave rattlesnakes without Mohave toxin are located in Arizona. Neurological toxicity is not observed in these bites.
• Systemic toxicity appears to be more severe in bites from Mohave rattlesnakes lacking Mohave toxin.

## Graphical abstract

**Figure:**
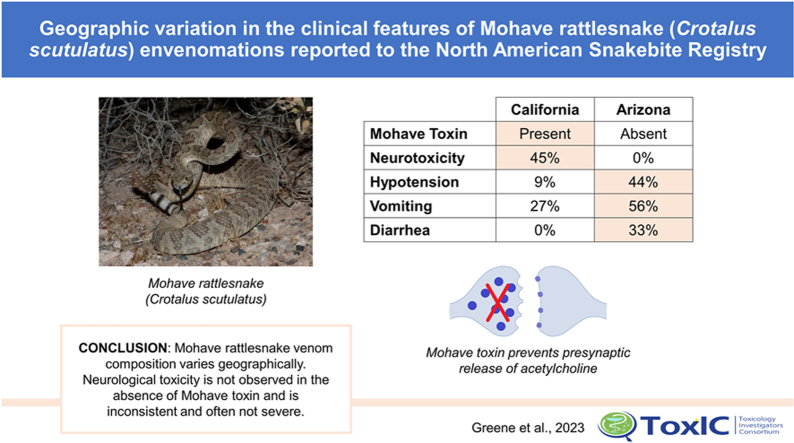

## 1 Introduction

The Mohave rattlesnake, *Crotalus scutulatus*, is a pitviper native to the Southwestern United States (U.S.) and Northern Mexico (Glenn and Straight, 1978). Although two subspecies were previously described, the Society for the Study of Amphibians and Reptiles (SSAR) has recognized only one species since 2017 (Bonett et al., 2017). Because “Mohave” is the spelling preferred by SSAR, it is used throughout this study.

Mohave rattlesnake envenomations are often described as being particularly dangerous because of a specific toxin found in their venom (Russell, 1969; Wingert and Chan, 1988; Ho and Lee, 1981). Mohave toxin is a neurotoxic phospholipase A2 that prevents the presynaptic release of acetylcholine, causing objective weakness that can progress to respiratory and skeletal muscle paralysis.

However, there is significant heterogeneity in Mohave rattlesnake envenomations. A retrospective study of 3440 rattlesnake envenomations in Arizona (AZ) between 1999 and 2020 found no cases of neurotoxic respiratory failure (Smelski et al., 2003). Only one patient in a review of 15 Mohave rattlesnake envenomations in AZ developed any neurological findings, and it was limited to mild eyelid ptosis (Hardy, 1983). Conversely, objective neurological deficits were reported in multiple patients in California (CA) (Farstad et al., 1997; Jansen et al., 1992).

The objective of this study was to describe the epidemiology, clinical effects, and management of Mohave rattlesnake envenomations, with an emphasis on the variation between two geographically distinct sites, using data reported to the North American Snakebite Registry (NASBR), administered by the American College of Medical Toxicology (ACMT).

## 2 Methods

The NASBR was established in 2013 as a sub-registry of the Toxicology Investigators Consortium (ToxIC) Core Registry, a voluntary, nationwide surveillance and research tool that prospectively records deidentified patient information from medical toxicologists providing bedside care for patients with a variety of toxicological exposures (Spyres et al., 2021). Details on snakebite data collection within the ToxIC NASBR have been reported previously (Ruha et al., 2017).

All ToxIC Core Registry and NASBR data were collected and managed by ACMT using Research Electronic Data Capture (REDCap) tools hosted at the Vanderbilt University Medical Center, Institute for Clinical and Translational Research. The ToxIC Core Registry and the NASBR are compliant with the Health Insurance Portability and Accountability Act and do not collect any protected health information or otherwise identifying fields. Registry participation is pursuant to the participating institutions’ Institutional Review Board (IRB) approval and compliance with their policies and procedures. The registries were also reviewed by the WCG IRB and determined not to meet the threshold of human subject research under federal regulation 45 CFR 46 and associated guidance.

For this report, all Mohave rattlesnake bites reported to NASBR from January 1, 2013 to December 31, 2021 were reviewed. Snake identifications were recorded by the treating medical toxicologist. Starting 2017, identifications were further classified as ***definite*** or ***likely***. Identification was considered definite if a snake expert directly visualized the snake (in person or by photograph) OR if there was only one snake endemic to a region. An identification was considered “likely” if it came from a witness with uncertain expertise in snake identification OR if an expert was forced to rely on photograph in which the snake was poorly visualized.

Local swelling was categorized as mild, moderate, or severe. Mild swelling was confined to <7 cm from the bite site. Moderate swelling extended 7–50 cm from the bite site or crossed a major joint. Swelling was considered severe if it extended >50 cm from the bite or beyond two major joints. Hypotension was defined as a systolic blood pressure <90 mm Hg. Neurological toxicity was classified as subjective paresthesias, objective weakness, and myokymia/fasciculations, which are not distinguished from each other in NASBR.

Data regarding the month and year of the snake encounter, patient demographics, state where treatment occurred, local and systemic signs and symptoms, antivenom utilization, other interventions, and outcomes were reviewed. Descriptive statistics were used to report results.

## 3 Results

There were twenty subjects, including eight (40%) non-pregnant females. All but two subjects were Caucasian. There was one Black patient and one for whom race was not recorded. Ages ranged from seven to 75 years, with a median age of 48 years old (IQR 30). Eleven (55%) bites were treated in southern California and nine (45%) bites were managed in central Arizona. Bites occurred from March through September, with the plurality of bites occurring in May. Month of bite was not available for three cases. All the bites occurred in the wild; none resulted from captive snakes. Patient characteristics are summarized in Table 1.

**Table 1:** Patient and snake demographics.

	Arizona	California
**# patients**	**9**	**11**
**Median age**	**52 (range: 8**–**75)**	**40 (range: 7**–**69)**
**Sex**		
• male	**5 (56%)**	**7 (64%)**
• female	**4 (44%)**	**4 (36%)**
**Bite location**		
• lower extremity	**5 (56%)**	**7 (64%)**
• upper extremity	**3 (33%)**	**4 (36%)**
• face	**1 (11%)**	**--**
**Intentional interaction with snake**	**3 (33%)**	**1 (9%)**
**Snake identification**		
• definite	**4 (44%)**	**2 (18%)**
• likely	**5 (56%)**	**6 (55%)**
• unavailable	**--**	**3 (27%)**

Definitive snake identification was possible for four (44%) AZ patients and two (18%) CA patients. In 11 cases, the snakes were “likely” *C. scutulatus*. Certainty of snake identification was not recorded for the three cases entered into NASBR before 2017.

Two (10%) patients had previous snakebites. One received antivenom for his previous snake envenomation. It is unknown if the other patient, who had two previous snakebites, received antivenom for either prior envenomation.

Twelve (60%) bites were to the lower extremity, seven (35%) bites occurred on the upper extremity, and there was one bite to the face after the victim intentionally played with the snake.

Four (20%) male patients intentionally interacted with the snake. Two had significant ethanol use just prior to getting bitten. One of these patients was bitten on the face. Another victim with evidence of recent alcohol consumption was bitten when he attempted to kick a snake and it became lodged in his footwear. A third subject picked up what he erroneously believed was a dead snake. The fourth patient was bitten when he attempted to remove the snake from a dog kennel.

Moderate swelling was most common, affecting nine (45%) patients. Four (20%) subjects had severe swelling, and mild swelling was observed in four (20%) others. No swelling was observed in three patients, including two who were deemed to have a dry bite. One CA patient who did not develop any swelling experienced pain and paresthesias consistent with a Mohave rattlesnake envenomation. Clinical features and hospital course are summarized in Table 2.

**Table 2:** Clinical features and hospital course.

	Arizona	California
Local swelling		
• none	**1 (11%)**	**2 (18%)**
• mild	**2 (22%)**	**2 (18%)**
• moderate	**4 (44%)**	**5 (45%)**
• severe	**2 (22%)**	**2 (18%)**
Thrombocytopenia	**1 (11%)**	**1 (9%)**
Hypotension	**4 (44%)**	**1 (9%)**
Emesis	**5 (56%)**	**3 (27%)**
Diarrhea	**3 (33%)**	**--**
Rhabdomyolysis	**--**	**2 (18%)**
Neurological toxicity	**--**	**5 (45%)**
Antivenom		
• none	**1 (11%)**	**1 (9%)**
• Fab	**5 (56%)**	**6 (55%)**
• F (ab’)2	**2 (22%)**	**2 (18%)**
• both	**1 (11%)**	**2 (18%)**
Hospitalization		
• < 24 h	**1 (11%)**	**3 (27%)**
• 24–48 h	**4 (44%)**	**5 (45%)**
• 49–72 h	**1 (11%)**	**1 (9%)**
• > 72 h	**3 (33%)**	**2 (18%)**
ICU admission		
• none	**1 (11%)**	**10 (91%)**
• < 24 h	**1 (11%)**	**--**
• 24–48 h	**4 (44%)**	**--**
• 49–72 h	**1 (11%)**	**1 (9%)**
• > 72 h	**2 (22%)**	**--**

Hematologic laboratory abnormalities were rare during initial hospitalization. There were no cases of hypofibrinogenemia or elevated prothrombin time. One AZ patient developed thrombocytopenia with a nadir of 78 K/mm^3^ platelets after receiving antivenom. A CA patient had a transient drop in platelets to 113 K/mm^3^ that normalized prior to discharge. Another CA patient without laboratory abnormalities had nuisance bleeding on initial presentation.

Hypotension was observed in five (25%) patients, including one patient prescribed an angiotensin-converting enzyme (ACE) inhibitor. Three of these patients were also tachycardic, and one, who was not on an ACE inhibitor, exhibited angioedema. Two patients taking an ACE inhibitor did not develop hypotension.

Eight (40%) patients developed emesis following the envenomation; five vomited within 1 h of the bite and before receiving opioids. An additional victim vomited 1–2 h after the bite but before being treated with opioids. Two others developed emesis after receiving opioids. Antiemetics were administered to 11 (55%) patients, including seven of the patients with emesis. Diarrhea was observed in three (15%) patients, including two patients who also vomited. One of these two was also taking an ACE inhibitor.

Rhabdomyolysis developed in two (22%) adult male CA patients, with peak creatine kinase (CK) measurements of 1393 IU and 5162 IU, respectively. Both had significant local swelling extending beyond two major joints. The patient with the peak CK of 1393 IU also had myokymia/fasciculations, emesis, perioral and extremity paresthesias, and transient mild thrombocytopenia.

Neurological toxicity was observed in five (25%) patients, all of whom were treated in California. Three patients reported only subjective paresthesias. Two patients experienced subjective paresthesias and myokymia/fasciculations. Paresthesias resolved following treatment with antivenom in one patient. It is unknown if antivenom reversed the symptoms in the other subjects.

One AZ patient with a history of dyslipidemia and hypertension, for which he was taking an ACE inhibitor, developed hypotension, tachycardia, diarrhea, and vomiting after sustaining a bite to the lower leg while walking his dog. He subsequently developed chest pain and was noted to have an ST elevation myocardial infarction. He underwent cardiac catheterization, which showed non-obstructive atherosclerotic disease in his left anterior descending artery as well as spasm of the right coronary artery. He was diagnosed with Kounis syndrome, in which mast cell activation leading to anaphylaxis manifests as vasospastic acute coronary syndrome (Kounis et al., 2011). This can occur with or without underlying coronary artery disease. In this case it was believed the patient had an allergic reaction to the rattlesnake venom, although the patient has no history of previous snakebite.

Antivenom was administered in 18 (90%) cases. The median time to antivenom was 3 h. Each site had one outlier; an AZ patient did not receive antivenom for 72 h, and one patient in CA was treated at 48 h. Eleven (55%) patients received only crotalidae polyvalent immune fab [ovine] (CroFab®, henceforth FabAV) and four (20%) were treated with crotalidae immune F (ab’)_2_ [equine] (Anavip®) exclusively. Three patients received both antivenom products. Two patients in CA and one AZ patient were given one antivenom after receiving the other product at the referring facility. There were no acute adverse reactions to either antivenom.

Hypotension was successfully treated with intravenous fluid resuscitation in two (40%) patients. Three (60%) patients also required vasopressors. An AZ patient who developed angioedema and hypotension following a bite to the face was intubated and placed on mechanical ventilation.

Antibiotics were administered empirically to two subjects with erythema and suspected cellulitis. A third patient received antibiotics for a confirmed soft tissue infection. No patients underwent debridement, dermotomy, or fasciotomy.

Patients were hospitalized for a median 24–48 h, with a range of <24 h to 12 days. Nine (45%) patients were admitted to the intensive care unit (ICU), with a median ICU length of stay (LOS) of 24–48 h. One (9%) CA patient and 18 (89%) AZ patients were admitted to the ICU.

Follow-up information was available for 9 (45%) patients: six (67%) AZ patients and three (27%) people treated in CA. Thrombocytopenia was present on first follow up for three patients, including one with thrombocytopenia while hospitalized. A fourth patient was found to be thrombocytopenic on the second follow up appointment. All patients with late thrombocytopenia received FabAV during their initial hospitalizations. No patient experienced bleeding. One patient was readmitted after sustaining head trauma in the setting of thrombocytopenia. One CA patient had serum sickness. One patient developed cellulitis at the IV site and was treated with oral antibiotics. Table 3 summarizes the unique clinical features and management of each subject.

**Table 3:** Individual patient demographics, clinical features, and treatment.

Age	Sex	Location	Snake identity confirmed	Bite site	Local findings	Vomiting, diarrhea	Hypotension	ACE-I use	Peak CK	Neurological toxicity	# vials AV	AV
8	F	AZ	Likely	Toe	–	–	–	–	n/a	–	–	–
39	M	AZ	Likely	Lower leg	Moderate	V D	IVF vasopressors	Y	n/a	–	18	F2
48	M	AZ	Definite	Face	Severe	V D	IVF vasopressors	–	373	–	24	F1 F2
48	M	AZ	Likely	Finger	Moderate	–	–	–	107	–	28	F1
52	F	AZ	Definite	Foot	Mild	V	–	–	n/a	–	4	F1
55	M	AZ	Definite	Lower leg	Mild	V	–	–	n/a	–	25	F1
63	F	AZ	Likely	Lower leg	Minimal	D	IVF vasopressors	–	n/a	–	18	F1
70	M	AZ	Likely	Hand	Moderate	V	IVF	–	69	–	22	F1
75	F	AZ	Definite	Hand	Moderate	–	–	–	n/a	–	24	F2

7	F	CA	Likely	Ankle	Moderate	–	IVF	–	203	–	20	F1 F2
9	F	CA	Definite	Ankle	Mild	V	–	–	n/a	–	44	F1 F2
27	F	CA	Likely	Foot	Moderate	–	–	–	341	P	20	F1
32	M	CA	Definite	Hand	Moderate	V	–	–	n/a	P M/F	33	F1
37	M	CA	Likely	Hand	Mild	–	–	–	300	–	20	F2
40	M	CA	Likely	Hand	Severe	V	–	–	1393	P	104	F1
45	M	CA	Likely	Foot	–	–	–	–	185	–	–	–
60	M	CA	n/a	Finger	Severe	–	–	–	5162	–	6	F1
60	F	CA	n/a	Toe	Moderate	–	–	Y	84	–	18	F1
67	M	CA	Likely	Foot	–	–	–	Y	290	P	6	F1
69	M	CA	Likely	Toe	Moderate	–	–	–	238	P M/F	10	F2

ACE-I = angiotensin-converting enzyme inhibitor.

CK = creatine kinase.

AV = antivenom.

V = vomiting.

D = diarrhea.

IVF = intravenous fluids.

n/a = not available.

F1 = Fab.

F2 = F(ab')2.

P = Paresthesias.

M/F = Myokymia/fasciculations.

## 4 Discussion

Mohave rattlesnakes are often cited as the most dangerous snakes in the U.S. However, relatively few deaths are reported. A review of 101 U.S. snakebite fatalities from 1989 to 2018 identified only four (4%) cases, including one suicide in which someone allowed his captive snake to bite him (Greene et al., 2021). An additional seven (7%) fatalities that were attributed to “unknown rattlesnake” occurred in lands inhabited by Mohave rattlesnakes as well as other rattlesnake species.

One explanation for the few deaths is the relative infrequency of Mohave rattlesnake bites. *Crotalus scutulatus* has a much smaller geographic distribution than many other species, and the areas where they reside are often sparsely populated. This may explain why species such as the timber rattlesnake (*C. horridus*) and eastern copperhead (*Agkistrodon contortrix*) are responsible for more deaths (Greene et al., 2021).

However, there is also significant geographic variability in Mohave rattlesnake venom composition. Type A Mohave rattlesnakes possess Mohave toxin but lack venom components found in other native rattlesnakes. These snakes are found in southern California, southern Nevada, southwest Utah, western New Mexico, southwestern Arizona, and southwestern Texas. Type B Mohave rattlesnakes lack Mohave toxin but do have the more common proteolytic and hemorrhagic activity. These snakes are confined to central Arizona. An intergrade zone exists in central Arizona. These snakes have been designated Type A + B because they possess both Mohave toxin and cytotoxic and hemotoxic venom components (Glenn and Straight, 1978, 1989). The subsequent discovery of a specific myotoxin (type M) in Mohave rattlesnakes in southeastern Arizona increased the number of Mohave phenotypes to six: A, A + M, B, B + M, A + B, A + B + M (Massey et al., 2012).

Mohave toxin is not unique to Mohave rattlesnakes, and multiple isoforms have been identified. A single isoform was isolated from the venom of five (20%) Southern Pacific rattlesnakes (*C. oreganus helleri*) found on Mt. San Jacinto in Riverside County, CA (French et al., 2004). Neurotoxicity has also been observed in envenomations from the midget faded rattlesnake (*C. concolor*), the western diamond-backed rattlesnake (*Crotalus atrox*), the tiger rattlesnake (*C. tigris*), the rock rattlesnake (*C. lepidus*) and the sidewinder (*C. cerastes*) (LoVecchio et al., 2005; Vohra et al., 2008; Bosak et al., 2014).

Our results confirm previous observations that neurological toxicity is uncommon in AZ Mohave rattlesnake envenomations. It was illuminating that neurotoxicity was seen in a minority of CA patients and that signs and symptoms were limited to paresthesias and myokymia/fasciculations. This may be due to timely administration of antivenom. Because FabAV contains antibodies specifically directed against *Crotalus scutulatus* venom antigens, effective neutralization would be expected. It is also possible that severe neurological manifestations occur relatively infrequently.

Hematologic toxicity was uncommon at both sites. Local damage and signs of systemic toxicity, including hypotension, vomiting, and diarrhea, were observed in both populations. There was a trend toward more significant hypotension and gastrointestinal effects in the AZ subjects, but the small sample size precludes meaningful statistical analyses.

There was great variability in the amount of antivenom administered. One pediatric subject received both products for a total of 44 vials despite having clinical features that were confined to vomiting and mild local swelling. Conversely, an adult male with rhabdomyolysis and severe local findings was treated with six vials of FabAV. Another adult male with severe local findings, vomiting, and mild rhabdomyolysis received 104 vials of FabAV. It is possible that multiple antivenom doses were given to patients prior to medical toxicology service involvement in the case.

**Fig. 1:**
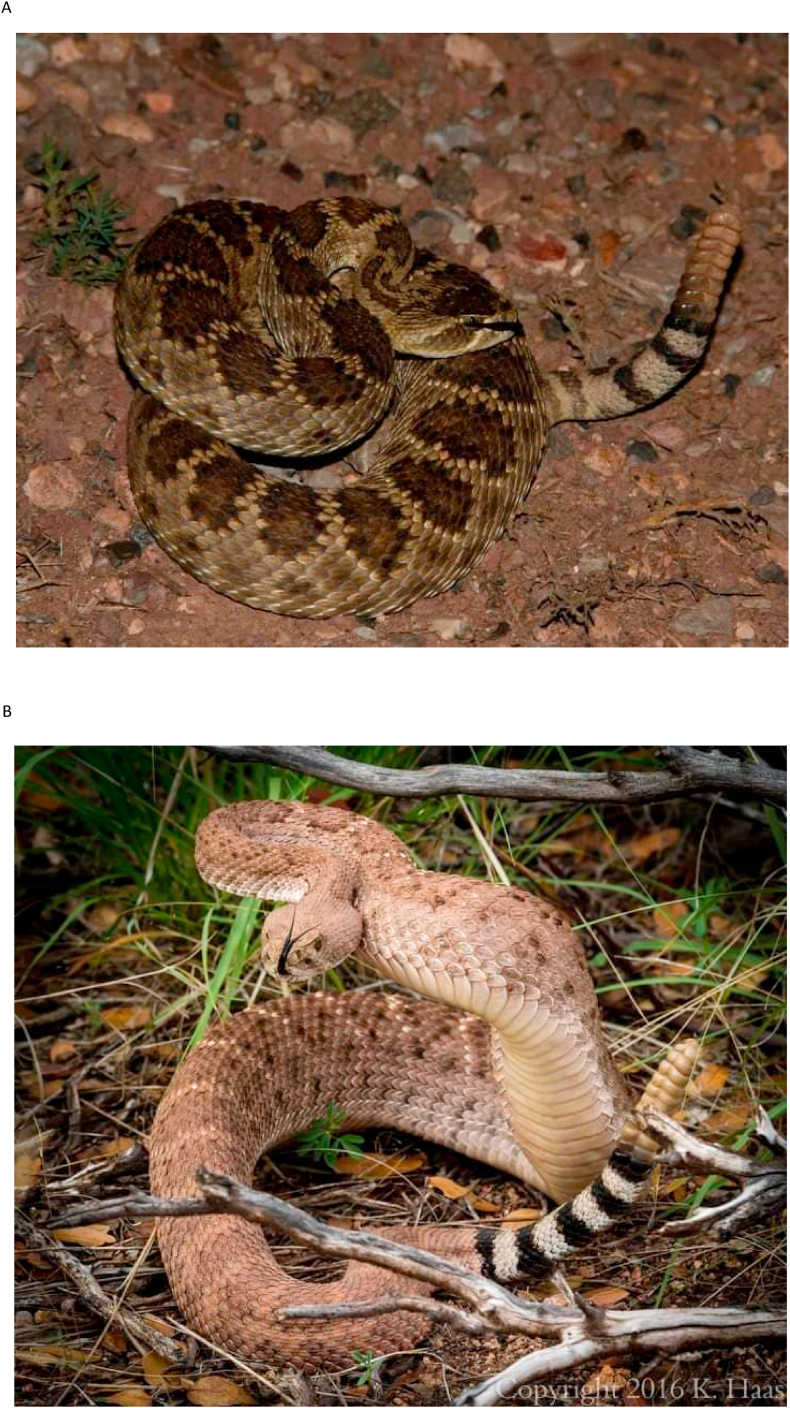
Comparison of Mohave rattlesnake (*Crotalus scutulatus*) and Western diamond-backed rattlesnake (*Crotalus atrox*) Fig. 1A. Mohave rattlesnake, *Crotalus scutulatus* Fig. 1B. Western diamond-backed rattlesnake, *Crotalus atrox*.

There are multiple limitations to the study. Only six (30%) snake identifications were considered “definite”. Mohave rattlesnakes resemble Western diamond-backed rattlesnakes (Fig. 1). Several physical characteristics (e.g., width of the white rings encircling the tail, the relationship of the postocular light stripe to the angle of the mouth, and the number of scales separating the suprocular scales), can be used to distinguish the two (Bush and Cardwell, 1999). Mohave rattlesnake may also occasionally be mistaken for other sympatric species, including the prairie rattlesnake (*C. viridis*), the eastern black-tailed rattlesnake (*C. ornatus*), and the western black-tailed rattlesnake (*C. molossus*) (Cardwell et al., 2022). The habitats of the Southern Pacific rattlesnake (*C. oreganus helleri*) and the Mohave rattlesnake also overlap. Because the Mohave toxin has been isolated from Southern Pacific rattlesnake venom, it is possible to confuse the species using clinical features.

The NASBR sites at which patients were treated may not accurately reflect the locations where the bites occurred; it is possible that the patients were transported from distant regions containing phenotypically different Mohave rattlesnakes.

Neurological toxicity is poorly characterized in this study. Three patients reported only paresthesias, but the quality and severity are not documented. Similarly, the extent of the fasciculations/myokymia observed in two cases is unclear.

Hospital and ICU LOS may not reflect the severity of envenomation. Many hospitals admit all snakebite patients to the ICU irrespective of severity. Others may be monitored in an ED observation unit. Length of hospitalization may have also been influenced by external factors, including, but not limited to, homelessness, access to follow up, and concomitant medical issues.

Finally, the small sample size precludes meaningful quantitative analysis. A study with more subjects would better distinguish actual geographic variations from random chance.

## 5 Conclusion

Neurotoxicity is not a feature of Mohave rattlesnake envenomation in AZ. Although neurotoxicity is observed in CA Mohave envenomations, it was limited to paresthesias and myokymia/fasciculations without objective weakness and respiratory failure in this study. Systemic toxicity, including gastrointestinal symptoms and hypotension, are common in AZ Mohave envenomations. Thrombocytopenia may be present during initial hospitalization or develop in a delayed fashion. Patients treated with FabAV are more likely to develop late thrombocytopenia. Finally, hypofibrinogenemia and coagulopathy are not common in Mohave envenomations in either CA or AZ. Larger studies involving more sites throughout the habitat of *C. scutulatus* are warranted to further elucidate the geographic variations of Mohave rattlesnake envenomations.

## Funding

The 10.13039/100011288ACMT North American Snakebite Registry is supported by an unrestricted grant to 10.13039/100011288ACMT from 10.13039/100014869BTG, International.

## Ethical statement

This study involved human subjects. The work has been carried out in accordance with The Code of Ethics of the World Medical Association. This is an observational study. Informed consent was obtained when patients sought treatment for their snakebites.

## CRediT authorship contribution statement

**Spencer Greene:** Writing – review & editing, Writing – original draft, Project administration, Formal analysis, Conceptualization. **Matthew Gilbert:** Writing – original draft, Formal analysis. **Brian Wolk:** Writing – review & editing, Data curation. **Sharan Campleman:** Project administration, Data curation. **Anne-Michelle Ruha:** Writing – review & editing, Investigation, Data curation.

## Declaration of competing interest

The authors declare that they have no known competing financial interests or personal relationships that could have appeared to influence the work reported in this paper.

## Data Availability

The authors do not have permission to share data.
